# Synthesis and Luminescent Properties of Europium Complexes Covalently Bonded to Hybrid Materials Based on MCM-41 and Poly(Ionic Liquids)

**DOI:** 10.3390/ma11050677

**Published:** 2018-04-26

**Authors:** Xiaolong Zheng, Meiyu Wang, Qiuping Li

**Affiliations:** School of Materials and Chemical Engineering, Ningbo University of Technology, No. 201, Fenghua Road, Jiangbei District, Ningbo 315211, China; Zhengxiaolong142@sina.com (X.Z.); Wangmeiyu130@163.com (M.W.)

**Keywords:** rare earth, ionic liquid, mesoporous material, hybrid material, luminescence

## Abstract

Due to the wide potential application in the fields of sensing, lighting materials, and optical–electrical multifunctional devices, rare earth complex hybrid materials have been studied extensively over the past decades. A poly(ionic liquid)/mesoporous-based hybrid system which has been functionalized by the covalently linked europium complexes was reported here. Through surface modification with a coupling agent bearing an vinyl group, MCM-41 was chosen as the carrier matrix for poly(ionic liquids) (PILs) and europium compounds, and based on that, novel luminescent hybrid materials were prepared by confining the ionic liquid and europium complexes into the inorganic Si–O frameworks. The resulting organic/inorganic materials are chemically bonded hybrids which show good photoluminescent properties such as broad excitation spectra, line-like emission spectra, and long luminescence lifetimes. The PILs/MCM-41/Eu^3+^ hybrid reported here is a rare earth multifunctional material which is believed to have potential applications in the field of optical–electrical materials.

## 1. Introduction

Because of their intense line-like luminesce emission and long lifetime, lanthanide complexes have been found to have diverse chemical and biological applications, such as light-emitting devices, luminescent optical fibers, fluorescent probes, and so on [1,2,3]. However, the poor stability of traditional lanthanide complexes has still limited their practical applications in some cases. Fortunately, the inserting of lanthanide complexes into various hybrid structures might offer an effective solution [4], which could be easily accomplished by the use of tailorable organic ligands [5,6,7]. Thus, the luminescent lanthanide hybrid materials have attracted much attention, owing to their powerful abilities of combining the outstanding photoluminescence of lanthanide elements with various matrices [8].

The important matrices for constructing luminescent lanthanide complex hybrid materials mainly include organic polymers [9], inorganic silica [10], inorganic zeolite [11], or inorganic–organic hybrid matrices and so on [12]. Recently, poly(ionic liquids) (PILs) have been found to have huge potential applications in some aspects of the material science field [13]. Since the ionic liquid was used for synthesizing lanthanide hybrid luminescent materials [14,15], many works have been focused on the using of PILs for lanthanide hybrid materials. Ru [16] has studied the synthesis of terpyridine moiety-modified PILs and their usage for constructing europium-based hybrid materials. Li [17] has reported the using of a bifunctional ionic liquid which carries a benzoic acid group and which acts as a matrix and second ligand simultaneously for preparing novel lanthanide complex functionalized hybrid PILs. The optical active hybrid materials based on PILs and inorganic matrices could find potential applications in the fields of optical–electrical devices such as organic light-emitting devices (OLEDs), luminescent solar concentrators, and so on [18,19]. However, the application of PILs for constructing inorganic–organic lanthanide complex hybrid materials has rarely been reported. Taking the covalent incorporation of europium complexes into the organic/inorganic matrices, here, the synthesis of novel luminescent rare earth complex functionalized materials based on the poly(ionic liquid)/mesoporous MCM-41 hybrid matrices has been discussed. The method reported here is a practice of constructing novel optical–electrical multifunctional materials, the functional moieties of which are linked covalently. Hence, a series of class II hybrid photoactive materials has been gained, which show good thermal stability and photoluminescence properties. Moreover, the resulting europium complex hybrid materials have been measured in detail with respect to its physical properties.

## 2. Materials and Methods

The 1-allyl-3-methylimidazolium chloride (AMIMCl) ionic liquid was purchased from Shanghai Cheng Jie chemical Co. LTD, while other reagents were provided by Aladdin reagents Co. LTD (Shanghai, China). The MCM-41 material was prepared according to the general procedures, and then was refluxed with ethanol in Soxhlet extraction for two days to remove the surfactant. After that, the MCM-41 was transferred into a solution of vinyltrimethoxysilane (VTMOS) in toluene and then refluxed at 80 °C for 12 h. The vinyl-functionalized MCM-41 was obtained from the solution and purified with toluene and ethanol, marked as MCM-41-Vinyl. The polymerizable europium complexes Eu(TTA)_3_L2 (TTA = 2-thenoyltrifluoroacetone, L2 = acrylic acid (AA), methacrylic acid (MAA), undecylenic acid (UA)) were synthesized by the well-established protocol [20]. The hybrid luminescent materials were prepared according to the following steps: A mixture of AMIMCl (1 mmol), Eu(TTA)_3_L2 (0.1 mmol), MCM-41-Vinyl (300 mg), and AIBN (10 mg) initiator were dispersed in dry N, *N*-Dimethylformamide (DMF, 10 mL) and sonicated for 30 min. The homogeneous solution was then heated to 70 °C with continuous stirring for 24 h under nitrogen atmosphere. The resulting solid material was collected by filtration and washed with ethanol and further purified by Soxhlet extraction with boiling ethanol for 48 h and finally dried in vacuum condition. The resulting materials were assigned as Eu@MCM-41-P (IL-AA), Eu@MCM-41-P (IL-MAA), and Eu@MCM-41-P (IL-UA), respectively. The hybrid materials were comprehensively characterized by XRD (Bruker D8, Ningbo University of Technology, Ningbo, China), FTIR spectroscopy (FTIR-8400S, Ningbo University of Technology, Ningbo, China), SEM (JEOL2011 microscope, Ningbo University of Technology, Ningbo, China), and TGA (Netzsch TG209F, Ningbo University of Technology, Ningbo, China) measurements. The photoluminescent behavior of materials was investigated by Hitachi F-4600 (Ningbo University of Technology, Ningbo, China) and Edinburgh Instrument FLS920 spectrophotometer (Tongji University, Shanghai, China). 

## 3. Results and Discussion

**Figure 1:**
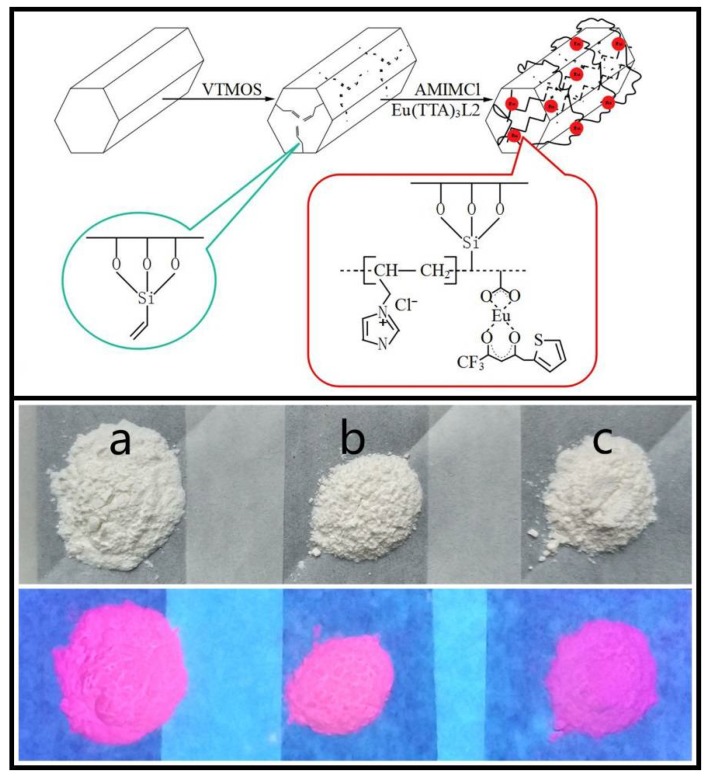
Synthetic scheme of the europium complex hybrid materials, and the photograph of the resulting materials under daylight (**top**) and 360-nm UV lamp (**bottom**). **a** = Eu@MCM-41-P (IL-AA); **b** = Eu@MCM-41-P (IL-MAA); **c** = Eu@MCM-41-P (IL-UA).

**Figure 2:**
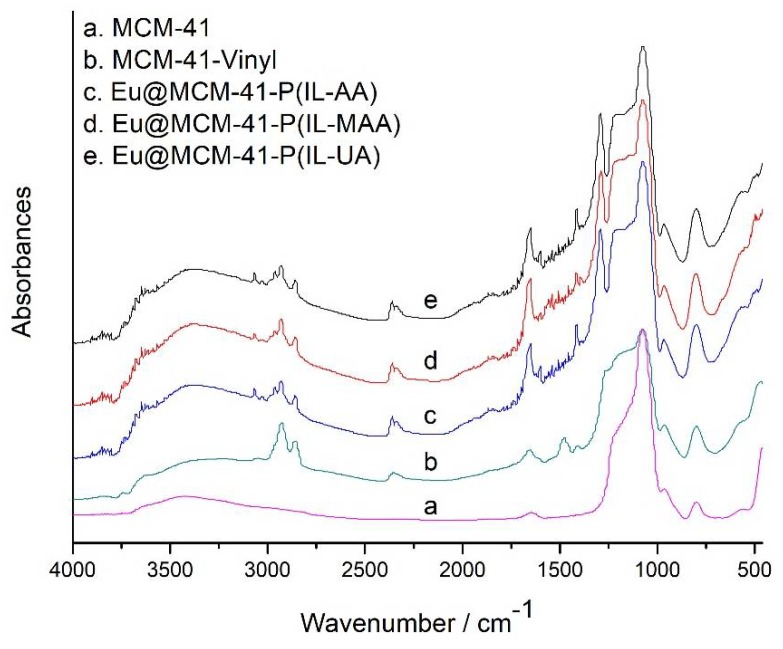
FTIR spectra of MCM-41, vinyl-modified MCM-41 (MCM-41-Vinyl), and the final materials.

The synthetic routes and schematic representation of europium complex hybrid materials are given in Figure 1. The digital photographs of powders of Eu@MCM-41-P (IL-AA), Eu@MCM-41-P (IL-MAA), and Eu@MCM-41-P (IL-UA) under daylight (top) and UV lamp (bottom) are also shown, and the characteristic emission of the Eu^3+^ ion was exhibited when exposed to UV light. The construction of hybrid materials was investigated by FTIR spectroscopy (Figure 2). As shown in the figure, the strongest broad bands appearing at about 1075 cm^−1^ in all spectra were assigned to Si–O–Si stretching vibrations of the mesoporous framework. The band at 1660 cm^−1^ of MCM-41-Vinyl was assigned to the stretching vibration of the C=C group, which meant that the surface modification of MCM-41 was successful. The absorption band at about 1575 cm^−1^ of the resulting hybrid materials (Figure 2c–e) were associated with the bending vibration of the imidazole ring that comes from the AMIMCl ionic liquid. In addition, the bands at 1600 cm^−1^ and 1415 cm^−1^ in the spectra of hybrid materials (Figure 2c–e) can be assigned to the asymmetric and symmetric vibrations of the COO group, respectively; and the bands at 1536 cm^−1^ can be assigned to the delocalisation of the π-electron in the chelate ring which forms between Eu^3+^ and the TTA molecules. These indicate that the europium complex functionalized poly(ionic liquid)/MCM-41 (PIL/MCM-41) hybrid materials had been successfully synthesized.

**Figure 3:**
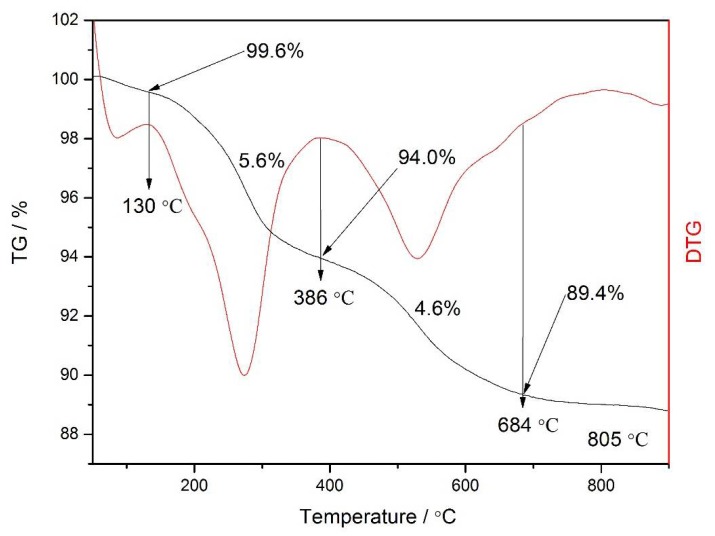
Thermogravimetric TG and derivative weight loss (DTG) curves of Eu@MCM-41-P (IL-AA).

The mesoporous structure of the hybrid materials has also been investigated and confirmed by X-ray diffraction (Figure S1, Supplementary Materials). The transmission electron microscope images (Figure S2, Supplementary Materials) could show the flaky structure of the hybrid material, but fail to reveal the details of the microstructure of MCM-41, since its surface was covered by the poly(ionic liquid). The thermal stability of the Eu@MCM-41-P (IL-AA) hybrid material was investigated and given as the representative. As shown in Figure 3, the thermogravimetric (TG) and the corresponding derivative weight loss (DTG) analysis revealed that the PIL/MCM-41-based hybrid materials showed a two-step weight loss approach, which was associated with the decomposition of polymer compositions attaching onto the exterior of MCM-41 and that of the one confined into the tunnel of MCM-41. Compared to the general decomposition temperatures of polymers, the hybrid materials possess a wider decomposition temperature interval, which can be seen as a sign of the improvement of the thermal stability.

**Figure 4:**
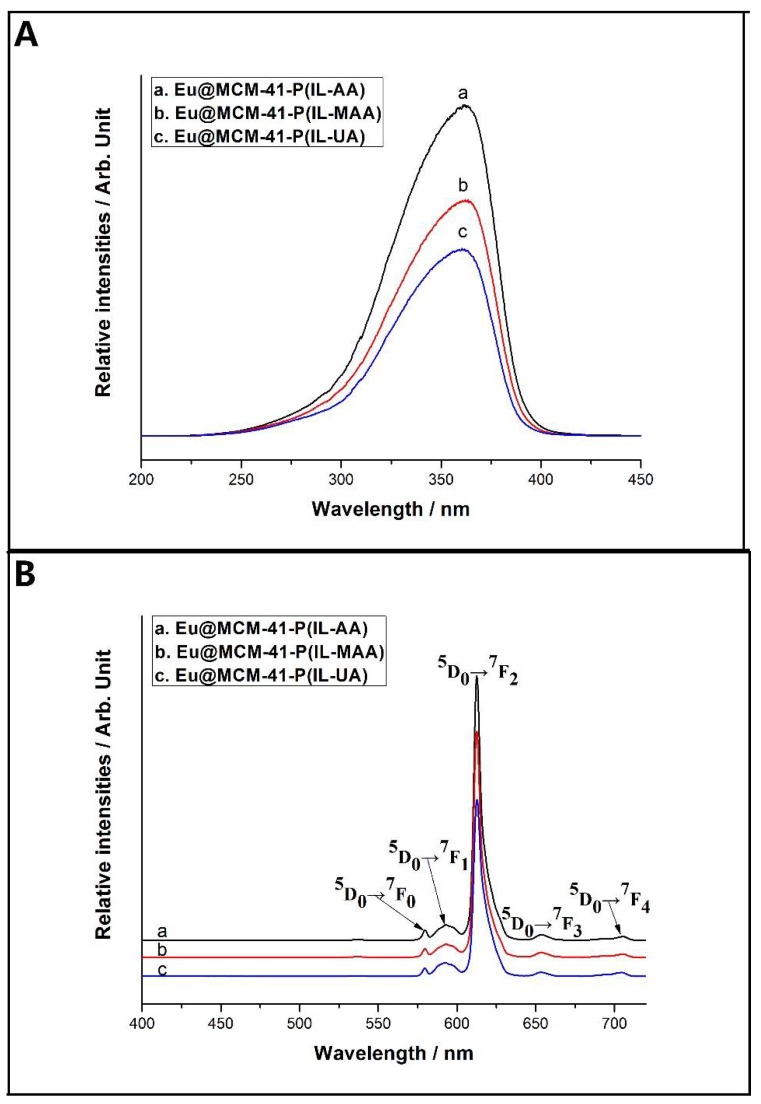
Excitation ((**A**) monitored at λ = 612 nm) and emission ((**B**) excited with λ = 360 nm) spectra of the hybrid materials.

The photoluminescent properties of the obtained hybrid materials have been investigated under room temperature. As shown in Figure 4A, the excitation spectra of Eu@MCM-41-P (IL-AA), Eu@MCM-41-P (IL-MAA), and Eu@MCM-41-P (IL-UA) were obtained by monitoring the characteristic emission ^5^D_0_→^7^F_2_ of the Eu^3+^ ion at 612 nm. It is clear from the spectra that all the hybrid materials have a broad band absorption in the range of 250–400 nm, which is ascribed to the absorption of the TTA ligand. The maximum peaks for hybrids center at about 360 nm, which is selected as the fixed excitation wavelength for measuring the emission spectra and luminescent lifetimes. As shown in Figure 4B, the emission spectra of all hybrid materials gave fine lines around 580, 593, 612, 653, and 703 nm, which could be assigned to the ^5^D_0_→^7^F_J_ (*J* = 0–4) transitions of the Eu^3+^ ion. All the emission spectrums were dominated by the very intense ^5^D_0_→^7^F_2_ transition line, which is responsible for the red luminescence emission of the hybrid materials.

**Figure 5:**
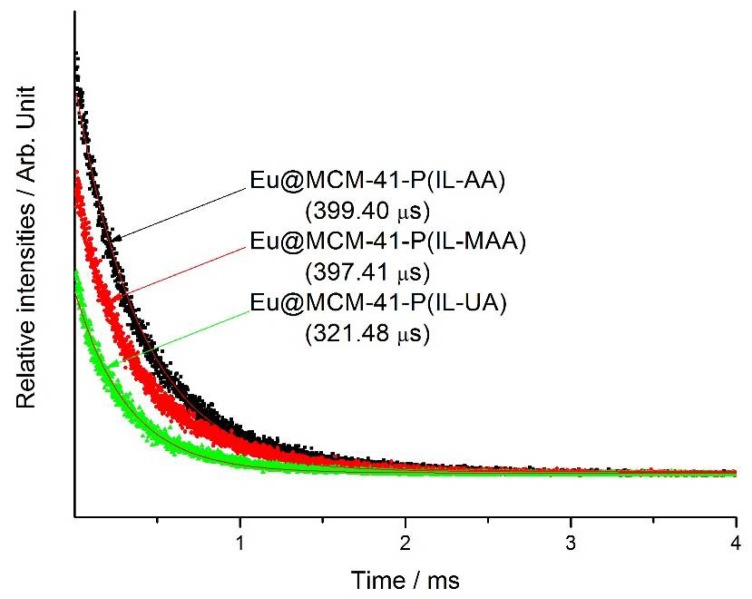
Luminescence decay curve for the ^5^D_0_→^7^F_2_ transition (612 nm) of the hybrid materials.

The luminescence decay properties of the hybrid materials shown in Figure 5 fitted well to a single exponential function. The decay times were calculated to be 399.40, 397.41, and 321.48 µs for Eu@MCM-41-P (IL-AA), Eu@MCM-41-P (IL-MAA), and Eu@MCM-41-P (IL-UA), respectively. Furthermore, based on the emission spectra and the lifetime (*τ*), the ^5^D_0_ quantum efficiencies of all the europium complex hybrid materials were estimated according to the Juud–Ofelt theory [21]. The detailed methods are listed below:

$$A_{0J}=A_{01}\times \frac{I_{0J}}{I_{01}}\times \frac{\nu_{01}}{\nu_{0J}}$$

$$A_{r}=\sum A_{0J}=A_{00}+A_{01}+A_{02}+A_{03}+A_{04}$$

$$\frac{1}{\tau }=A_{r}+A_{nr}$$

$$\eta =\frac{A_{r}}{A_{r}+A_{nr}}$$

Here, *A*_01_ is Einstein’s coefficient of spontaneous emission between ^5^D_0_ and ^7^F_1_, the value of which is theoretically determined to be 50 s^−1^; *I*_0*J*_ is the ^5^D_0_→^7^F*_J_* (*J* = 0~4) transition intensities calculated from the peaks; and the *ν*_0*J*_ (*J* = 0~4) is the energy barycenter of the corresponding emission peaks. Moreover, the *A_r_* is the rate of radiative transition, while *A_nr_* is that of nonradiative transition. Based on these, the luminescence quantum efficiencies of europium complex hybrid materials can be seen as equal to the ratio of *A_r_* to the value of (*A_r_* + *A_nr_*). The obtained luminescence data are summarized in Table 1. All the luminescent quantum efficiencies of the hybrid materials are over thirty percent, as shown in the table, and it is clear that Eu@MCM-41-P (IL-AA) (37.1%) and Eu@MCM-41-P (IL-MAA) (36.1%) have similar values, higher than that of Eu@MCM-41-P (IL-UA) (33.2%), which is consistent with the result of luminescence lifetimes. From these results, it is reasonable to assume that the second ligand (i.e., UA) with a long chain structure works against the photoluminescent properties. On the other hand, the reason why the hybrid materials Eu@MCM-41-P (IL-AA) and Eu@MCM-41-P (IL-MAA) show very similar luminescence quantum efficiencies and lifetimes is probably due to having similar molecular structures to those of AA and MAA ligands.

## 4. Conclusions

In summary, a series of europium-based hybrid materials Eu@MCM-41-P (IL-L2) (L2 = AA, MAA, UA) was prepared successfully through an in situ polymerization of polymerizable europium complexes, polymerizable ionic liquids, and vinyl-modified MCM-41. The resulting luminescent materials are chemically bonded organic/inorganic hybrids that could be excited by 360-nm light effectively and exhibited strong red emission mainly originating from the ^5^D_0_→^7^F_2_ transition (612 nm) of Eu^3+^ ions. The materials have good thermal stability and outstanding photoluminescent properties. The luminescent quantum efficiencies of Eu@MCM-41-P (IL-AA) and Eu@MCM-41-P (IL-MAA) are very close and higher than that of Eu@MCM-41-P (IL-UA), apparently indicating that a smaller second ligand would beneficial for the photoluminescent properties of the europium complex hybrid materials reported here. The present investigation manifested that the PILs/MCM-41 hybrid is a good matrix for constructing novel rare earth hybrid materials. Future works will focus on its application in optical/electrical fields.

## Figures and Tables

**Table 1:** Luminescent data for the hybrid materials.

	*τ* (μs) ^a^	*A_r_* (s^−1^)	*A_nr_* (s^−1^)	*η* (%) ^b^
Eu@MCM-41-P (IL-AA)	399.4	928.0	1573.3	37.1
Eu@MCM-41-P (IL-MAA)	397.4	907.1	1605.6	36.1
Eu@MCM-41-P (IL-UA)	321.5	1031.7	2075.8	33.2

^a^ lifetimes (*τ*) of the ^5^D_0_ energy level of the Eu^3+^ excited state; ^b^ the luminescent quantum efficiency (*η*) value of the ^5^D_0_ Eu^3+^ excited state calculated from decay times and emission intensities.

## Supplementary Materials

The following are available online at http://www.mdpi.com/1996-1944/11/5/677/s1: Figure S1: XRD patterns of MCM-41 and the final materials; Figure S2: TEM of the Eu@MCM-41-P (IL-AA).
