# Supplementary material for: Synthesis and Luminescent Properties of Europium Complexes Covalently Bonded to Hybrid Materials Based on MCM-41 and Poly(Ionic Liquids)
Source: Materials (Basel). 2018 Apr 26;11(5):677. doi: 10.3390/ma11050677 (PMC5978054; doi:10.3390/ma11050677)
Supplement: Supplementary file 1 [file materials-11-00677-s001.pdf]

materials

Supplementary Materials

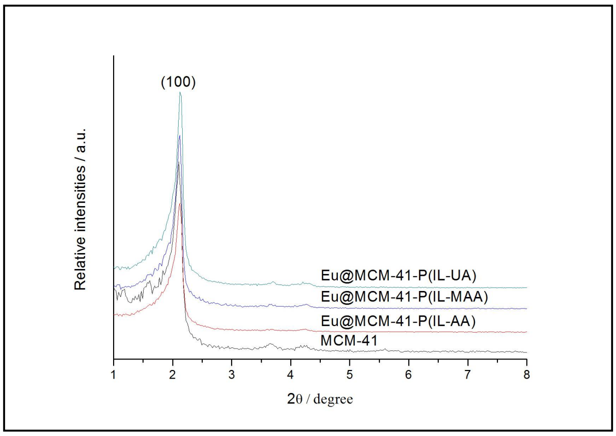

Figure S1. XRD patterns of MCM-41 and the final materials.

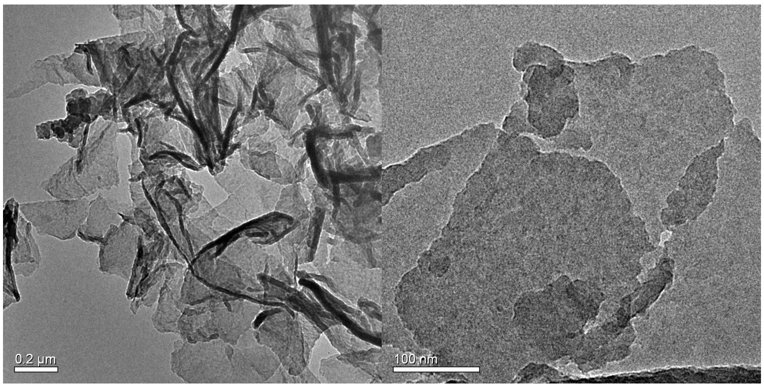

Figure S2. TEM of the Eu@MCM-41-P(IL-AA).
